# Circulating microRNA signatures for diagnosis and prediction of curve progression in pediatric patients with idiopathic scoliosis

**DOI:** 10.1186/s13018-025-06614-1

**Published:** 2026-01-05

**Authors:** Jana Orlickova, Michael Lujc, Michal Galko, Dagmar Al Tukmachi, Ondrej Slaby, Martin Repko

**Affiliations:** 1 Department of Experimental Biology, Faculty of Science, Masaryk University, Brno, Czech Republic; 2 Department of Biology, Faculty of Medicine, CEITEC - Central European Institute of Technology, Masaryk University, Brno, Czech Republic; 3 Department of Orthopedics and Spine Surgery, Faculty of Medicine, University Hospital Brno, Masaryk University, Brno, Czech Republic

**Keywords:** Idiopathic scoliosis, Progression risk, MicroRNA profiling, NGS, Biomarker study

## Abstract

**Background:**

Idiopathic scoliosis (IS) is the most common pediatric spinal deformity, yet no biomarker currently enables early diagnosis or reliable prediction of progression to guide individualized treatment. Circulating microRNAs (miRNAs) are promising non‑invasive biomarkers reflecting multifactorial disease mechanisms.

**Methods:**

In our prospective monocentric study, a Czech cohort comprising 114 pediatric IS patients at the time of diagnosis and 89 age‑matched healthy controls was studied. Risk groups were defined based on the final Cobb angle at the end of follow-up at skeletal maturity. Plasma miRNA profiles were obtained by small RNA sequencing and analyzed for differential expression. Logistic regression models were used to construct miRNA diagnostic and prognostic signatures, validated by leave‑one‑out cross‑validation (LOOCV).

**Results:**

Differential expression analysis identified 48 miRNAs with significantly different expression in the blood plasma of IS patients and controls (adj. *p* < 0.05), and plasma miR-4451 to have decreased levels in high-risk compared to low- and medium-risk IS patients (adj. *p* < 0.01). A 28‑miRNA diagnostic signature distinguished IS patients from controls with AUC = 0.95 (sensitivity 88%, specificity 92%) and LOOCV accuracy = 0.85. For prognosis, comparison of high‑risk versus low/medium‑risk patients revealed a 7‑miRNA prognostic signature, achieving AUC = 0.83, sensitivity 82%, specificity 74% and LOOCV accuracy = 0.81. Notably, the incorporation of clinical variables such as age or sex did not improve significantly model performance.

**Conclusions:**

Our study highlights the clinical utility of miRNA‑based models for precise diagnosis and individualized patient management and supports further validation in larger, independent cohorts.

**Supplementary Information:**

The online version contains supplementary material available at 10.1186/s13018-025-06614-1.

## Introduction

A child’s growth can be affected by the development of scoliosis. Scoliosis is a complex three-dimensional deformity of the spine in the frontal, sagittal, and axial planes, most often of unknown origin, that typically occurs in pediatric patients between 10 and 16 years of age [1]. Up to 80% of scoliosis cases are of unknown etiopathology, i.e., idiopathic scoliosis (IS). IS affects around 2% of children [2], with the most severe cases occurring predominantly in girls, at a ratio of up to 10:1 [1]. This orthopedic condition impacts not only the musculoskeletal system but also the development of thoracic and abdominal organs, potentially leading to a significant reduction in quality of life and impaired social integration in affected children.

Regarding treatment options, the current gold standard in idiopathic scoliosis consists of regular medical check-ups of the spine using X-ray imaging to monitor potential progression of the disease by measuring the Cobb angle. This approach, however, often results in a significant delay of clinical intervention, as treatment is usually initiated only once substantial curve progression has been documented. Additional limitations include considerable interindividual variability in Cobb angle measurements, which may introduce bias, and the absence of a fully standardized methodology, leading to inconsistencies in treatment decisions across different clinicians and centers. To date, no clinical biomarkers are available for the early detection of IS progression.

IS is considered a complex disease with marked genetic heterogeneity, and numerous theories of its etiopathogenesis and progression have been proposed, including disturbances in growth, energy metabolism, hormonal regulation, and genetic factors [3, 4]. The often ambiguous results of these studies have shifted attention toward factors that may reflect both the patient’s external and internal environment [5, 6].

At present, the most recent studies focus either on epigenetic signatures of IS-associated genes, primarily DNA methylation profiles [7–10] or on microRNA (miRNA) levels in body fluids and scoliosis-related tissues. MicroRNAs are small, non-coding RNAs that regulate gene expression at the post-transcriptional level and play key roles in many biological processes. Dysregulation of non-coding RNAs and mainly miRNAs has been linked to various musculoskeletal disorders and related pathological processes, including rheumatoid arthritis [11, 12], osteoporosis [13–16], tendon injuries [17, 18], bone fracture healing [19], spinal tuberculosis [20], and intervertebral disc degeneration [21, 22]. But more Importantly, recent miRNA studies have demonstrated a mechanistic involvement of miRNAs in IS pathogenesis and also their potential utility as diagnostic and prognostic biomarkers in IS patients [23–32].

Our study focused on circulating plasma miRNAs and their potential as diagnostic biomarkers and indicators of IS progression. A crucial part of the study was comparing circulating miRNA levels in the blood plasma of high-risk patients with those of low and medium-risk risk to identify prognostic biomarkers enabling the prediction of curve progression in IS patients.

## Methods

### Patient cohort and controls

The study cohort was recruited at our center and comprised 114 patients with idiopathic scoliosis (IS) and 89 healthy controls. Patients were followed for 24 months. Plasma samples were collected from all participants for subsequent miRNA analysis. All study participants provided written informed consent. An overview of the cohort characteristics is provided in Tables 1 and 2.

All patients enrolled in the study, both female and male, were diagnosed with either juvenile or adolescent idiopathic scoliosis. At baseline, the main structural curve measured between 16° and 40°, with a Risser sign of 0–3. Patients were regularly monitored throughout the observation period, and all were treated with corset therapy. IS patients were stratified into three risk subgroups according to the final Cobb angle at the end of the follow-up (low risk ≤ 25 °, medium risk > 25° ≤ 35°, high risk ≥ 35 °). Patients were regularly monitored throughout the observation period, and those whose curve magnitude exceeded 20° during follow-up were prescribed brace treatment with a recommended minimum wear time of 16 h per day.

Exclusion criteria for our study included Risser sign 4–5, skeletal maturity, curve less than 10 or more than 40 degrees requiring surgery, more than two years post-menarche, cases of non-idiopathic scoliosis (e.g., neuromuscular, congenital, or other causes), and patients with intellectual disability or other cognitive impairments.

**Table 1:** Characterization of patient cohort

Patients (*n* = 114)			
Variable	Mean [95% CI]	Median [95% CI]	Range
Age	12.4 [12.1–12.8]	12.9 [12.6–13.2]	7–15
Initial Cobb Angle	24.8 [23.5–26.0]	24.0 [23.0–26.0]	11–39
Final Cobb Angle	29.6 [27.4–31.8]	29.0 [26.0–32.0]	5–57
BMI	17.8 [17.45–18.2]	17.6 [17.5–17.6]	13–26
*Gender*			
Female		100 (88%)	
Male		14 (12%)	
*Risk subgroups*			
Low risk		35 (31%)	
Medium risk		35 (31%)	
High risk		44 (38%)	
*Menses status*			
Premenarche		60 (60%)	
Postmenarche		40 (40%)	
*Diagnosis*			
Juvenile		49 (43%)	
Adolescent		65 (57%)	

CI * Confidence interval*, BMI * Body mass index*

**Table 2:** Characterization of control cohort

Controls (*n* = 89)			
Variable	Mean [95% CI]	Median [95% CI]	Range
Age	12.0 [11.7–12.4]	11.9 [11.5–12.4]	8–16
BMI	18.4 [18.0- 18.88]	18.3 [18.23–18.35]	12.4–24.7
*Gender*			
Female		66 (74%)	
Male		23 (26%)	
*Menses status*			
Premenarche		39 (59%)	
Postmenarche		24 (36%)	
NA		3 (5%)	

CI *Confidence interval*, BMI *Body mass index*, NA * Not available*

### Circulating MiRNA expression profiling

Approximately 5 mL of peripheral venous blood was collected in an ethylenediaminetetraacetic acid (EDTA)-treated Vacutainer. Within one hour after collection, plasma fraction was separated by centrifugation at 2000× g for 10 min at 4 °C and stored at − 80 °C till further processing. RNA was extracted using the miRNeasy Serum/Plasma Kit (Qiagen, USA) according to the manufacturer’s instructions and stored at ‑80 °C. cDNA libraries were prepared using the QIAseq^®^ miRNA UDI Library Kit (Qiagen, USA). The quantity and quality of the libraries were checked by Qubit^®^ 4.0 Fluorometer (Invitrogen, USA) and Tape Station 2200 System (Agilent, USA). Sequencing analysis was performed using the NovaSeq 6000 S2 v1.5 Kit (100 cycles) on the NovaSeq 6000 instrument (both Illumina, USA). Three sequencing runs were performed. Analysis of the miR-451a/miR-23a ratio in our sequencing data, following the methodology of Smith et al., did not reveal any signs of haemolysis in our blood plasma samples, with all values below the established threshold (median ratio 0.9, range 0.2–1.4) [33].

### Bioinformatic analysis

Raw sequencing data were converted to FASTQ format using bcl2fastq (v2.20.0) and quality-checked with FASTQC (v0.11.8). Adapter sequences were identified with Kraken (15–065) and trimmed with Cutadapt (v3.3) allowing a 10% error rate. Reads were collapsed using unique molecular identifiers (UMIs) with FASTX-Toolkit (v0.0.14); UMIs were subsequently trimmed with Cutadapt and reads shorter than 15 bp were discarded. Reads were checked for contamination using STAR (v2.7.0d). Surviving reads were mapped to miRBase v22 and quantified with miraligner tool seqcluster (v1.2.8), seqbuster (v3.5) and isomiRs (v1.22.0). miRNAs with fewer than 10 counts in at least 10% of samples were filtered out. Differential expression analysis was performed on normalized data using DESeq2 (v1.30.1) and adjusted to covariates such as age, menarche, sequencing run, and progression risk. In total, 652 miRNAs were detected in our cohort. Our data are publicly available under the GEO accession number GSE308158 [34]. Significantly dysregulated miRNAs and their target genes were analysed using functional pathway analysis based on the Gene Ontology (GO) and Kyoto Encyclopaedia of Genes and Genomes (KEGG) databases.

### Statistical analysis

All data were statistically evaluated using R v4.0.4 and GraphPad Prism 8. Different risk groups of IS patients (high, medium, low) were compared in terms of their demographic characteristics (age, BMI, gender, menses status, height). This evaluation did not reveal any significant differences in demographic parameters among the different risk groups of IS patients except height (*p* = 0.04 between high- and medium-risk groups). Nonparametric tests were applied (Kruskal–Wallis, Chi-square, Mann–Whitney, and Fisher’s exact test).

A univariate logistic regression approach was applied to the construction of miRNA signatures. Data were first corrected for batch effects using the ComBat function in R and normalized with DESeq2. Only miRNAs with at least 10 reads per million in at least 50 samples were retained (354 in total). A univariate logistic regression approach was applied to pre-select candidates for miRNA signatures, and the Benjamini-Hochberg correction was used to obtain adjusted p-values. Bidirectional stepwise selection was applied to identify the most suitable model and ROC curves were generated using significance thresholds of *p* < 0.1 or *p* < 0.15. The maximum Youden index was used to obtain an optimal cut-off value for the discrimination of the groups. Finally, model performance was validated using leave-one-out cross-validation (LOOCV).

## Results

In our monocentric prospective biomarker study, we enrolled 114 IS patients and 89 healthy controls (Tables 1 and 2). The cohort comprised both juvenile idiopathic scoliosis (JIS) and adolescent idiopathic scoliosis (AIS) patients, since most previous studies have focused exclusively on the AIS subgroup. To broaden the scope, we deliberately enriched our cohort with JIS patients. In addition, as male patients are rarely included in similar research, we incorporated them to assess potential sex-related differences in IS risk and progression.

### Diagnostic MiRNAs

Although our primary focus was on prognostic biomarkers, we first explored the diagnostic potential of circulating miRNAs in our cohort. Comparison of IS patients and healthy controls revealed 48 significantly dysregulated miRNAs (adjusted *p* < 0.05). In the comparison of high-risk patients versus controls, 19 miRNAs were significantly dysregulated, with 7 miRNAs (miR-223-3p, miR-320c, miR-320d, miR-766-3p, miR-1908-5p, miR-3124-5p, and miR-6515-5p) being specific to the high-risk IS subgroup (Table 3).

**Table 3:** Dysregulated MiRNAs with diagnostic potential in our cohort

IS patients vs. controls				high-risk IS patients vs. controls			
miRNA	log2FC	*p* value	adj.* p* value	miRNA	log2FC	*p* value	adj.* p* value
**miR-182-5p**	0.7983	0.0000	0.0000	**miR-182-5p**	0.9088	0.0000	0.0001
**miR-12,136**	− 0.7395	0.0000	0.0000	**miR-12,136**	− 0.8510	0.0000	0.0001
**miR-125b-5p**	0.5134	0.0000	0.0001	**miR-6802-5p**	− 0.6692	0.0000	0.0044
**miR-150-5p**	0.5434	0.0000	0.0003	**miR-125a-5p**	0.3800	0.0000	0.0044
**miR-320a-3p**	− 0.2436	0.0000	0.0003	**miR-1275**	− 0.5313	0.0000	0.0044
miR-451a	0.7565	0.0000	0.0004	**miR-125b-5p**	0.4731	0.0001	0.0118
**miR-1275**	− 0.4367	0.0000	0.0006	**miR-320a-3p**	− 0.2465	0.0002	0.0126
miR-144-3p	0.6767	0.0000	0.0009	**miR-326**	0.5382	0.0002	0.0126
**miR-130b-3p**	− 0.2280	0.0000	0.0014	**miR-130b-3p**	− 0.2491	0.0003	0.0147
miR-106b-5p	0.3375	0.0001	0.0025	**miR-760**	− 0.3345	0.0003	0.0147
**miR-183-5p**	0.6220	0.0001	0.0025	**miR-6764-5p**	− 0.5505	0.0004	0.0177
miR-664a-5p	− 0.2202	0.0001	0.0037	miR-6515-5p	− 0.3818	0.0005	0.0192
**miR-6802-5p**	− 0.5062	0.0001	0.0037	**miR-5187-5p**	− 0.3547	0.0006	0.0238
miR-30b-3p	− 0.2521	0.0001	0.0043	**miR-6803-3p**	0.8376	0.0008	0.0262
miR-130a-3p	− 0.2227	0.0002	0.0049	**miR-532-3p**	0.4713	0.0009	0.0285
miR-92a-3p	0.3316	0.0002	0.0049	**miR-150-5p**	0.4456	0.0011	0.0323
**miR-5187-5p**	− 0.3062	0.0002	0.0060	**miR-3200-5p**	1.0398	0.0012	0.0323
**miR-532-3p**	0.4008	0.0002	0.0060	miR-320c	− 0.3223	0.0012	0.0323
**miR-760**	− 0.2794	0.0002	0.0060	**miR-183-5p**	0.5811	0.0014	0.0346
**miR-6764-5p**	− 0.4424	0.0003	0.0062	miR-1908-5p	− 0.3403	0.0016	0.0346
**miR-125a-5p**	0.2626	0.0003	0.0066	**miR-3123**	− 0.4724	0.0014	0.0346
miR-29b-3p	0.3245	0.0003	0.0067	miR-320d	− 0.3196	0.0015	0.0346
**miR-3123**	− 0.4542	0.0004	0.0078	miR-3124-5p	− 0.4621	0.0019	0.0392
miR-660-5p	0.2908	0.0004	0.0083	miR-766-3p	0.4826	0.0019	0.0392
miR-1273 h-5p	− 0.2533	0.0007	0.0131	miR-223-3p	0.2018	0.0021	0.0408
miR-15a-5p	0.3487	0.0007	0.0132	**miR-484**	0.4509	0.0023	0.0434
miR-12,135	− 0.6255	0.0008	0.0133				
miR-4451	0.9165	0.0009	0.0159				
let-7e-5p	− 0.2792	0.0010	0.0159				
miR-10b-5p	0.3600	0.0011	0.0159				
**miR-3200-5p**	0.8053	0.0011	0.0159				
miR-3615	0.3585	0.0011	0.0159				
**miR-6803-3p**	0.5831	0.0011	0.0159				
miR-7-5p	0.2795	0.0011	0.0159				
miR-6886-5p	− 0.6905	0.0013	0.0170				
miR-151a-5p	− 0.1817	0.0016	0.0209				
**miR-326**	0.3072	0.0020	0.0253				
miR-3613-5p	0.5272	0.0023	0.0285				
**miR-484**	0.3163	0.0025	0.0305				
miR-30c-1-3p	− 0.2240	0.0028	0.0331				
miR-4665-5p	− 0.2535	0.0029	0.0341				
miR-6892-5p	− 0.5587	0.0033	0.0372				
miR-331-3p	0.4022	0.0036	0.0398				
miR-197-3p	0.2398	0.0039	0.0419				
let-7d-3p	0.2301	0.0040	0.0421				
miR-16-5p	0.2249	0.0043	0.0443				
miR-1180-3p	0.3651	0.0044	0.0445				
miR-128-3p	0.1394	0.0050	0.0495				

miRNAs significant in both comparisons are bolded. Log2FC – log2 fold change. Bold miRNAs indicate overlap between both comparisons

### Prognostic MiRNAs

The primary aim of our study was to investigate the prognostic potential of circulating miRNAs as biomarkers of IS progression. In the comparison of high-risk versus low-risk patients, miR-4451 was significantly differentially expressed (Log2FC = − 7.4, adj. *p* < 0.0008). The same miRNA was also the only one significantly dysregulated when high-risk patients were compared with the combined group of low- and medium-risk patients (Log2FC = − 0.003, adj. *p* < 0.003).

### Diagnostic and prognostic MiRNA signatures

**Fig. 1:**
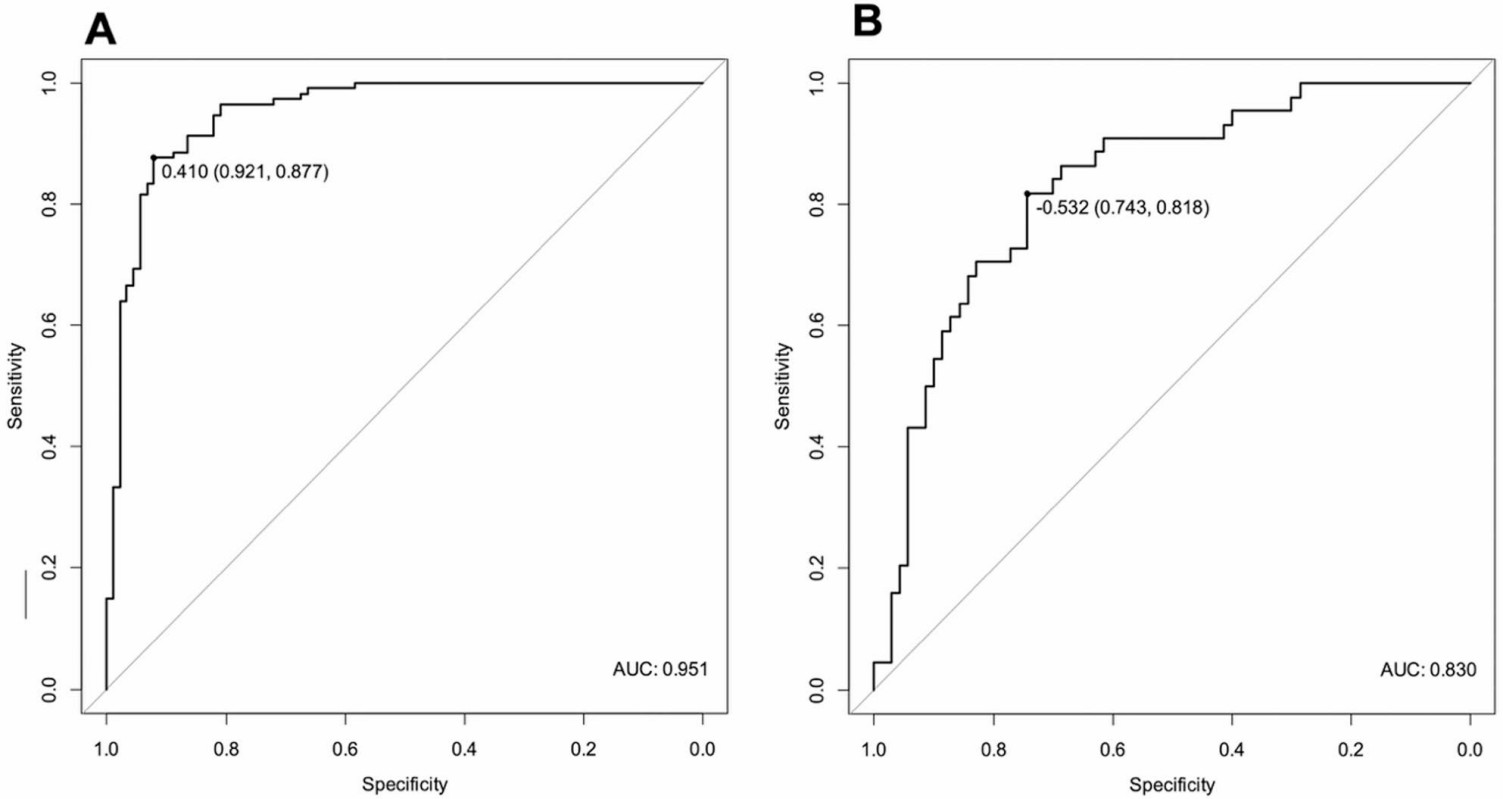
Receiver operating characteristic (ROC) curves of the diagnostic **A** and prognostic miRNA signatures **B**. AUC – Area under curve

Direct comparisons between patient risk subgroups did not yield satisfactory results; therefore, we pre-selected miRNAs with univariate logistic regression (*p* < 0.1 and OR > 1), then applied bidirectional stepwise selection followed by multivariate logistic regression to construct multi-miRNA signatures capable of distinguishing IS patients from controls as well as among risk groups. Established DiagnosticScore, based on the expression levels of 28 miRNAs, achieved an AUC of 0.95 (95% CI: 0.92–0.98, sensitivity 89%, specificity 92%) and a cross-validation accuracy of 0.85 (Table 4; Fig. 1A).

We then performed logistic regression analysis to distinguish between risk subgroups, focusing on the comparison of high-risk versus low/medium-risk patients, as our primary interest was to differentiate high-risk cases. The best model for PredictiveScore, based on the expression levels of 7 miRNAs, achieved an AUC of 0.83 (95% CI: 0.75–0.91, sensitivity 82%, specificity 74%) with a cross-validation accuracy of 0.81 (Table 5; Fig. 1B).

Notably, adding additional parameters to our models, including several clinical variables such as height, BMI, sex, menses status, did not improve their performance.

**Table 4:** Characteristics of the 28-miRNA diagnostic signature based on receiver operating characteristic (ROC) analysis and LOOCV validation

	IS patients vs. controls (28-miRNA diagnostic signature)
DiagnosticScore formula	DiagnosticScore= − 174.6996320 + 2.1746097*let-7d-3p + 0.5411538*miR-106b-3p + 1.6271858*miR-106b-5p + 3.1004664*miR-10b-5p − 1.4529877*miR-1180-3p + 1.5749691*miR-1226-3p + 0.6780959*miR-1304-5p − 0.5086980*miR-145-5p − 0.5636765*miR-183-5p − 2.6701470*miR-20b-5p + 5.4584333*miR-223-3p − 8.7724369*miR-25-3p − 2.2606958*miR-29b-3p + 0.9171286*miR-301a-3p + 4.7356280*miR-30e-5p + 0.5383158*miR-3157-5p − 0.7375481*miR-328-3p + 1.1372469*miR-331-3p − 2.2017905*miR-339-5p + 1.2877046*miR-4433b-5p + 2.3247527*miR-451a + 2.8585071*miR-4732-5p − 0.4579905*miR-501-3p +0.8779461*miR-589-5p + 2.2074434*miR-660-5p + 4.7794010*miR-7-5p + 0.8955283*miR-93-3p + 1.2636502*miR-942-5p
DiagnosticScore cut-off	0.41 (higher values indicate IS patients)
AUC [95% CI]	0.95 [0.92–0.98]
Sensitivity [95% CI]	0.878 [0.82–0.93]
Specificity [95% CI]	0.92 [0.87–0.98]
MSE	0.15
PPV	0.93
NPV	0.85
Cross-validation accuracy	0.85

AUC * Area under curve*, CI * Confidence interval*, MSE * Mean standard error*, PPV/NPV * Positive/negative predictive value*, LOOCV * Leave‑one‑out cross‑validation*

**Table 5:** Characteristics of the 7-miRNA predictive signature based on receiver operating characteristic (ROC) analysis and LOOCV validation

	High risk vs. Low + medium risk IS patients (7-miRNA signature)
PredictiveScore formula	PredictiveScore = − 7.1195743 + 0.2584089*miR-136-3p + 0.3589880*m iR-183-3p + 0.7946402*miR-361-3p − 0.7155679*miR-375-3p + 0.6794575*miR-548am-5p − 0.7710385*miR-627-5p + 1.2799934*miR-941
PredictiveScore cut-off	− 0.53 (higher values indicate High Risk)
AUC [95% CI]	0.83 [0.75–0.91]
sensitivity [95% CI]	0.82 [0.71–0.93]
specificity [95% CI]	0.74 [0.64–0.84]
MSE	0.19
PPV	0.67
NPV	0.87
Cross-validation accuracy	0.81

AUC * Area under curve*, CI * Confidence interval*, MSE * Mean standard error*, PPV/NPV * Positive/negative predictive value*, LOOCV * Leave‑one‑out cross‑validation*

### Other statistical comparisons

In addition to tracking IS progression in our overall cohort, we explored potential differences between IS subtypes and other clinically relevant factors, such as menses status. IS can be classified by age of onset into infantile, juvenile, and adolescent forms. Since most studies focus exclusively on AIS, we sought to investigate possible variability between the two most common subgroups, JIS and AIS. However, we found no significant differences in miRNA expression between them, suggesting comparable miRNA profiles. We identified significantly dysregulated miRNAs when comparing male IS patients with male controls (Supplementary Table S1) and risk-subgroups (high- versus low- and medium-risk), with fold changes of a reasonable magnitude (Supplementary Table S2). We did not identify any significant differences in miRNA levels when directly comparing male and female patients. Next, we compared patients by menarche status, both between IS patients and controls and within IS patients, and observed some differences, although the log2FC values were modest. Moreover, the sets of dysregulated miRNAs identified in IS patients and controls were nearly identical, except for three miRNAs (Supplementary Table S3).

### Functional pathway analysis

Even though our main goal was to seek biomarkers of IS progression, we also conducted pathway analysis to determine the biological significance of predicted mRNA targets of identified diagnostic and prognostic miRNAs in IS pathology (Supplementary Fig. S1, S2, S3 and S4). Although many of the predicted mRNAs are involved in processes such as muscle tissue development, organ growth, regulation of developmental growth, or mesenchyme development, it remains very difficult to unequivocally link these findings to the pathogenesis of idiopathic scoliosis. This is largely due to the limited current understanding of IS pathophysiology and the potentially pleiotropic effects of the identified miRNAs.

## Discussion

For many decades, researchers have attempted to clarify the etiopathology of idiopathic scoliosis, which has proven to be a considerable challenge. To date, numerous lines of research have investigated the origins of IS, often yielding inconclusive results. Studies have ranged from analyses of serum levels of various compounds (such as hormones, vitamins, or minerals) related to bone remodeling, growth, or energy metabolism, to association studies of single nucleotide polymorphisms (SNPs). More recently, attention has shifted toward epigenetic markers, which may better capture the complexity of IS [5].

Equally important as understanding the etiology of IS is elucidating its progression, which is crucial for patient treatment strategies. Beyond regular imaging check-ups, there are essentially no alternatives for monitoring IS progression. Repeated X-ray screening, however, is invasive and problematic for the developing child. Moreover, assessment of the Cobb angle, Risser sign, or other clinical markers relies heavily on the subjective judgment of the examining orthopedist. This has motivated the search for more objective and less invasive biomarkers. Unfortunately, serum and genetic markers have not proven helpful. For example, the ScoliScore test [35], based on 53 SNPs and Cobb angle values, was developed in a U.S. population but failed validation in Japanese [36], French Canadian [37], and Han Chinese [38] cohorts, highlighting population specificity.

Similarly, individual miRNA levels do not appear to serve as universal biomarkers of IS or curve progression. While other groups have also reported some of the dysregulated miRNAs identified in our study—for instance, upregulation of miR-15a-5p in IS patients versus controls (also seen in Li’s study [28]) or upregulation of miR-223-3p in high-risk patients versus controls (also reported in a Spanish cohort [24])—overall overlap between studies remains limited. In certain cases, we observed concordance with additional miRNAs, such as miR-766-3p or miR-1180-3p (reported in an Italian cohort), but these were not among the top markers in other studies. We also found consistent dysregulation within the miR-30 family, though different members (miR-30b-3p and miR-30c-1-3p). This observation is noteworthy, as the Italian study detected these miRNAs in both plasma and extracellular vesicles (EVs), a biological source increasingly explored in biomarker research [30, 39].

Our subgroup analyses yielded several noteworthy findings. No significant differences were observed when patients were stratified by age. However, we detected subtle but significant alterations in the miRNA profiles of female patients in relation to their menarche status. In contrast, the male subgroup appeared more distinctive, as we identified significantly dysregulated miRNAs when comparing male IS patients with male controls and risk-subgroups (high- versus low- and medium-risk) of male IS patients, with fold changes of a reasonable magnitude. On the other hand, when directly comparing male and female patients, we did not detect any significant differences in miRNA levels. It is important to note that our subgroup analyses were performed on small datasets, and therefore our results require validation in larger male cohorts. Nonetheless, focusing on male patients may provide valuable insights into both the onset and progression risk of IS. Of all previously published miRNA studies, only three included male patients [24, 27, 30]. The Spanish cohort was analysed only as a whole, while the Canadian study identified sex-specific differences in miRNA profiles. Moreover, significant alterations were observed in Canadian males when moderate progressors were compared to non‑progressors. Both phenomena were also detected in our cohort, as well as in the Italian study.

Another important aspect is the analytical performance of individual miRNAs and combined miRNA signatures. Four research teams have evaluated the analytical performance of the miRNA/miRNAs to predict curve progression. The best model reported by Khatami et al. [27] used six miRNAs to distinguish high-risk from low-risk patients and achieved an AUC of 1; however, such analytical performance has not been observed in any other study. Chen et al. [26] evaluated miR-96-5p alone, with an AUC of 0.671. Adding additional parameters, including age, years since menarche, and bone mineral density of various regions, improved the AUC to 0.82. Two further studies evaluated analytical performance, one based on a single miRNA (miR-151a-3p), achieving an AUC of 0.885 [31], and another using four miRNAs with an AUC of 0.95 (specificity 0.90, sensitivity 0.857) [24]. In our study, the 7-miRNA signature reached an AUC of 0.83.

It is important to note that most published studies compare severe IS patients with healthy controls to identify ‘progression’ biomarkers. In this study, patients were stratified into three risk categories (low-risk, medium-risk, and high-risk) based on the Cobb angle measured at the last follow-up. This stratification reflects both the therapeutic principles applied within each category and the risk of further curve progression after skeletal maturity, while also accounting for the interindividual variability of manual Cobb angle measurement, reported in the literature to reach up to 5° [40]. The first, low risk group, with Cobb angle < 25° comprised patients managed with physiotherapy, modification of lifestyle measures, and, in selected cases, brace treatment. A Cobb angle of 30° has been reported in the literature as a threshold below which long-term health consequences in adulthood, such as back pain or further deformity progression, are rarely observed [41]. On the other hand, in curves exceeding 30° of scoliosis, the likelihood of curve progression in adulthood increases, accompanied by an increased risk of health complications and a potential decline in quality of life [42]. Ohashi et al. report based on a 25-year follow-up of the ThL curves an average of 37.3 ± 7.5 degrees at the time of skeletal maturity, subsequent progression of 0.5 degrees per year [43]. The same authors reported over 40% of the curves reaching > 50° during the mean 25-year follow-up period with a median Cobb angle of 36.5° at skeletal maturity [44]. Alcala et al. state in their study that all AIS curves between 30° and 50° at skeletal maturity tend to progress. Thoracic curves progress more than lumbar curves during the first 20 years, and then progression slows down [45]. Dragstedet et al. in their study with a 40-year follow-up showed that mean progression for 25–40° curves at skeletal maturity was 16° [46]. The threshold for operative treatment of scoliosis varies internationally and regionally, ranging between 40 and 50 degrees of the Cobb angle for posterior spinal fusion. However, some novel non-fusion techniques, like anterior vertebral body tethering, have enabled early surgical management of the growing spine, which can be indicated for curves with a Cobb angle as low as 35° [47]. Therefore, curves exceeding 35° of Cobb angle are considered a high-risk group for the purpose of this study. The medium-risk group, defined by a Cobb angle of 25–35°, represents a “gray zone” between low- and high-risk categories when considering the known variability of radiographic measurements. From a therapeutic perspective, this group is clinically important because it includes patients who are managed conservatively, with physiotherapy and bracing, and who do not yet fulfil the established criteria for surgical intervention.

In addition, not all dysregulated miRNAs in these studies reached an adjusted *p* < 0.05, and the cohorts were smaller and composed exclusively of AIS patients. Taken together, it is therefore unsurprising that our results demonstrate more continuous values and subtler differences across comparisons. Nevertheless, we believe that stratifying patients into subgroups and comparing them with each other, rather than only with healthy controls, provides a more accurate representation of the progression process. In this context, the performance of our models is comparable to those reported by other studies.

Our study has several limitations. A limitation of our study is the imperfect matching of patients and controls with respect to sex. Unfortunately, we were not able to assemble a control cohort composed almost exclusively of girls within the given timeframe. The sequencing data were partly affected by a batch effect, and although we applied the best possible correction, validation in an independent cohort is required. We identified a few miRNAs that were significantly dysregulated in similar studies on IS; nevertheless, these findings need to be confirmed in further research. Even though this represents a significant improvement over the current state, PredictiveScore’s analytical performance is still insufficient: with a PPV of 0.67, 33% of patients classified as ‘high risk’ would in fact be false positives, which could lead to unnecessary stress and overtreatment, such as unwarranted bracing. This limitation could potentially be addressed by using combined models, for example, by integrating miRNA profiles with DNA methylation profiles, which may improve overall predictive accuracy. Future validation should also extend beyond the Czech population to better assess the universality of this type of biomarker.

Finally, the differences detected in our datasets were subtle, which may reflect the complex nature of IS as a disease. We identified differentially expressed miRNAs in IS patients compared with healthy controls and developed a 28-miRNA diagnostic signature enabling the diagnosis of IS patients from blood plasma specimens with a validated accuracy of 85%. However, when IS patients were compared according to progression risk status, variability was even lower, and no meaningful changes in miRNA expression were found. Similarly, no major differences were observed between JIS and AIS subgroups. Delayed menarche has traditionally been considered a clinical biomarker of IS severity [48], and our results provide some support for this theory, although validation in an independent cohort is necessary. Importantly, our study also included male patients, who are rarely represented in this research area.

## Conclusions

To our knowledge, this is the first study to compare miRNA levels not only between IS patients and healthy controls but also within IS subgroups to enable the prediction of IS progression at the time of diagnosis. Although we did not identify any individual miRNA that could serve as a reliable biomarker of IS progression in the Czech population, we were able to construct 7-miRNA predictive signature that distinguished high-risk patients from other risk groups with a validated accuracy of 81%.

## Supplementary Information

Below is the link to the electronic supplementary material.

Supplementary Material 1.

## Data Availability

RNA sequencing data are available in NCBI GEO repository here: [https://www.ncbi.nlm.nih.gov/geo/query/acc.cgi? acc=GSE308158](https:/www.ncbi.nlm.nih.gov/geo/query/acc.cgi? acc=GSE308158).

## Abbreviations

AIS: Adolescent idiopathic scoliosis
AUC: Area under curve
BMI: Body mass index
cDNA: Complementary DNA
CI: Confidence interval
EDTA: Ethylenediaminetetraacetic acid
IS: Idiopathic scoliosis
JIS: Juvenile idiopathic scoliosis
Log2FC: Fold change of logarithm with a base of two
LOOCV: Leave‑one‑out cross‑validation
miRNA: MicroRNA
MSE: Mean standard error
NA: Not available
NPV: Negative predictive value
PPV: Positive predictive value
ROC: Receiver operating characteristics
SNP: Single nucleotide polymorphism
