# Supplementary material for: Circulating microRNA signatures for diagnosis and prediction of curve progression in pediatric patients with idiopathic scoliosis
Source: J Orthop Surg Res. 2026 Jan 5;21:88. doi: 10.1186/s13018-025-06614-1 (PMC12870172; doi:10.1186/s13018-025-06614-1)
Supplement: Supplementary file 1 — Supplementary Material 1. [file 13018_2025_6614_MOESM1_ESM.docx]

**Supplementary material**

Table S1 Dysregulated miRNAs with diagnostic potential in male IS patients.

| **miRNA** | **log2FC** | **p value** | **adj. p value** |
| --- | --- | --- | --- |
| miR-451a | 1,7051 | 0,0000 | 0,0005 |
| miR-182-5p | 1,2158 | 0,0000 | 0,0007 |
| miR-10b-5p | 0,8599 | 0,0002 | 0,0096 |
| miR-1246 | 0,8599 | 0,0002 | 0,0096 |
| miR-7-5p | 0,7433 | 0,0001 | 0,0096 |
| miR-1180-3p | 0,8657 | 0,0003 | 0,0129 |
| miR-125b-5p | 0,6934 | 0,0005 | 0,0129 |
| miR-15a-5p | 0,7589 | 0,0004 | 0,0129 |
| miR-16-5p | 0,6800 | 0,0003 | 0,0129 |
| miR-183-5p | 1,0585 | 0,0004 | 0,0129 |
| miR-144-3p | 1,1043 | 0,0006 | 0,0142 |
| miR-25-3p | 0,4767 | 0,0010 | 0,0223 |
| miR-4732-5p | 0,7186 | 0,0015 | 0,0315 |
| miR-20b-5p | 0,6916 | 0,0016 | 0,0318 |
| miR-29b-3p | 0,6109 | 0,0020 | 0,0360 |
| miR-34a-5p | 0,4627 | 0,0025 | 0,0420 |
| miR-92a-3p | 0,4720 | 0,0030 | 0,0479 |

*Log2FC – log2 fold change.*

Table S2 Dysregulated miRNAs with predictive potential in male IS patients (high- versus low- and medium-risk).

| **miRNA** | **log2FC** | **p value** | **adj. p value** |
| --- | --- | --- | --- |
| miR-28-3p | 1,5691 | 4,78E-07 | 4,42E-05 |
| miR-625-5p | 1,5723 | 4,40E-07 | 4,42E-05 |
| miR-11400 | 3,4686 | 1,17E-05 | 0,0007 |
| miR-92a-3p | -1,1909 | 0,0002 | 0,0081 |
| miR-486-5p | -1,3722 | 0,0007 | 0,0272 |
| miR-223-5p | 0,9234 | 0,0013 | 0,0411 |

*Log2FC – log2 fold change.*

Table S3 Dysregulated miRNAs in comparisons based on menarche status (on the left in IS patients, on the right in IS patients and controls).

| **premenarche vs. postmenarche IS cases** | | | | **premenarche vs. postmenarche controls** | | | |
| --- | --- | --- | --- | --- | --- | --- | --- |
| miRNA | log2FC | p value | adj. p value | miRNA | log2FC | p value | adj. p value |
| miR-149-5p | -0,1565 | 0,0008 | 0,0469 | miR-193a-5p | 0,1103 | 0,0000 | 0,0003 |
| miR-181c-5p | -0,0733 | 0,0009 | 0,0469 | miR-193b-5p | 0,0556 | 0,0000 | 0,0003 |
| miR-193a-5p | 0,0540 | 0,0000 | 0,0013 | miR-3131 | -0,3232 | 0,0000 | 0,0025 |
| miR-193b-5p | 0,0011 | 0,0000 | 0,0005 | miR-320b | 0,1295 | 0,0004 | 0,0187 |
| miR-3131 | -0,1868 | 0,0000 | 0,0028 | miR-320c | 0,0988 | 0,0002 | 0,0103 |
| miR-320b | 0,0635 | 0,0005 | 0,0381 | miR-483-5p | 0,0811 | 0,0008 | 0,0322 |
| miR-320c | 0,0398 | 0,0002 | 0,0153 | miR-487b-3p | -0,1562 | 0,0005 | 0,0205 |
| miR-483-5p | -0,0579 | 0,0001 | 0,0131 | miR-92b-5p | 0,1667 | 0,0001 | 0,0103 |
| miR-92b-5p | 0,1053 | 0,0000 | 0,0011 |  |  |  |  |

*Log2FC – log2 fold change.*

**Supplementary Figures**

**
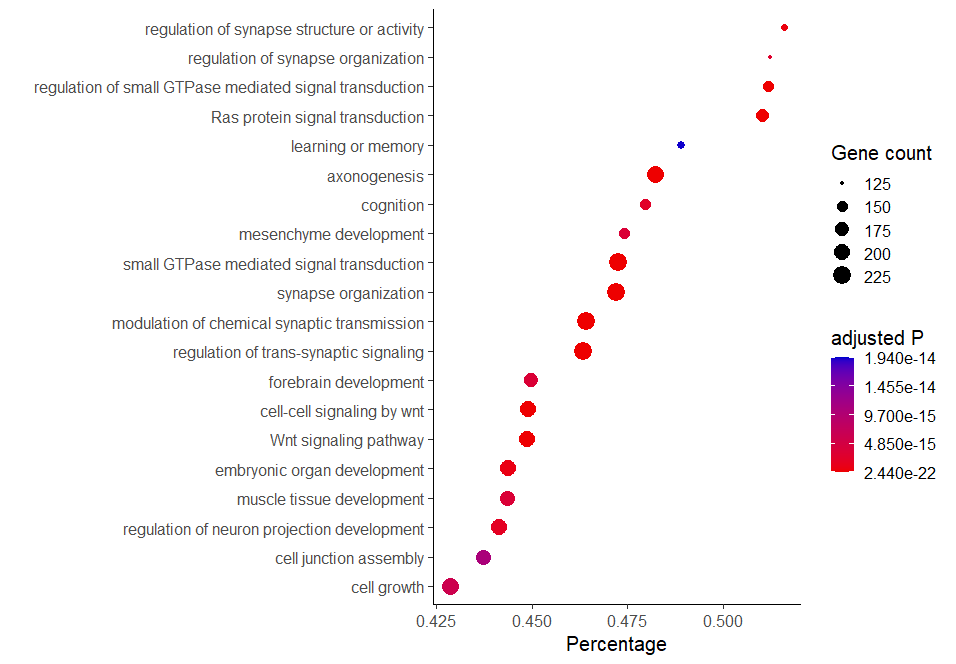
**

¨

Figure S1: Molecular functions associated with predicted target genes of dysregulated miRNAs used for DiagnosticScore using GO (Gene Ontology) database analysis.


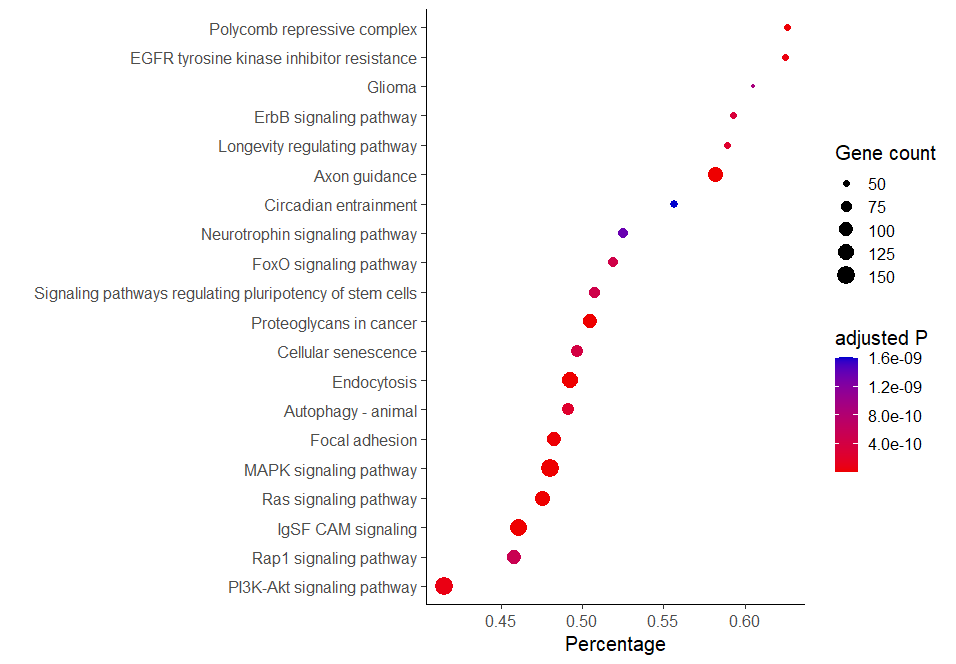


Figure S2: Biological pathways associated with predicted target genes of dysregulated miRNAs used for DiagnosticScore based on KEGG database analysis.


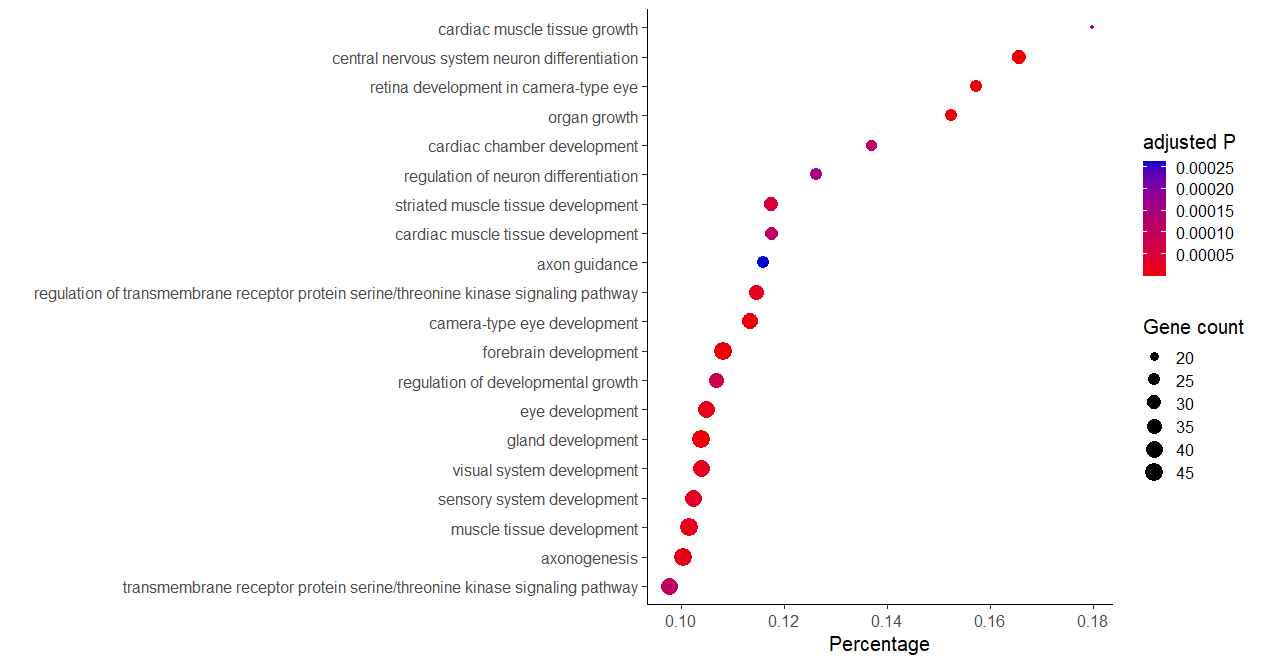


Figure S3: Molecular functions associated with predicted target genes of dysregulated miRNAs used for PredictiveScore using GO (Gene Ontology) database.


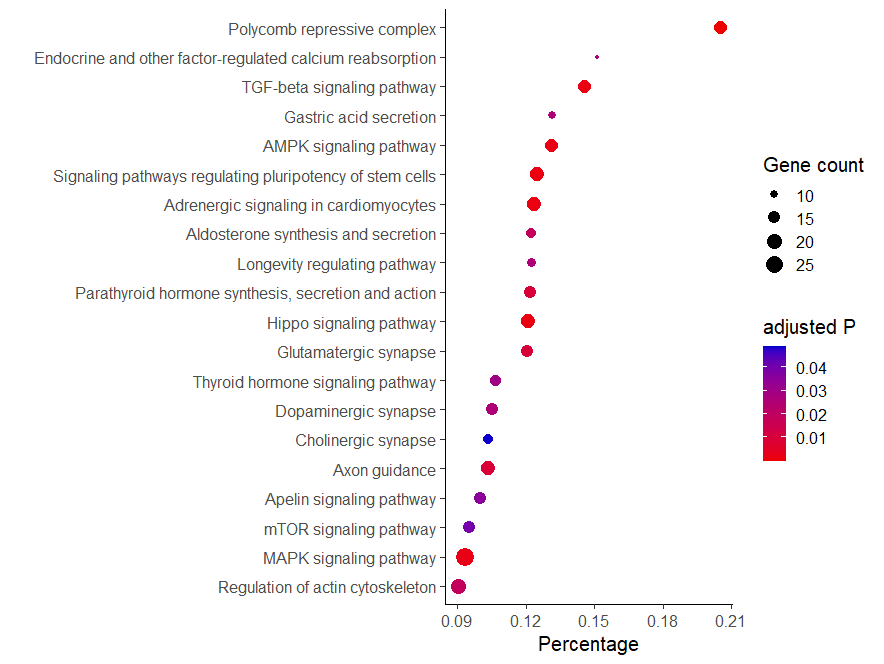


Figure S4: Biological pathways associated with predicted target genes of dysregulated miRNAs used for PredictiveScore based on KEGG database analysis.
